# Resolved Proteinuria May Attenuate the Risk of Heart Failure: A Nationwide Population-Based Cohort Study

**DOI:** 10.3390/jpm13121662

**Published:** 2023-11-28

**Authors:** Yoonkyung Chang, Min Kyoung Kang, Moo-Seok Park, Gwang-Hyun Leem, Tae-Jin Song

**Affiliations:** 1 Department of Neurology, Mokdong Hospital, Ewha Womans University College of Medicine, Seoul 08209, Republic of Korea; ykchang@ewha.ac.kr; 2 Department of Neurology, Seoul Hospital, Ewha Womans University College of Medicine, Seoul 07804, Republic of Korea; yen101@ewha.ac.kr (M.K.K.); strokesolved@ewha.ac.kr (M.-S.P.); 3 Ewha Medical Research Institute, Ewha Womans University, Seoul 07804, Republic of Korea; shalomlkh@ewha.ac.kr

**Keywords:** proteinuria, heart failure, dipstick test, urinalysis, epidemiology

## Abstract

Although proteinuria is a risk factor for heart failure (HF), proteinuria can be reversible or persistent. Our objective was to explore the link between changes in the proteinuria status and the risk of HF. We included participants from a Korean national health screening cohort who underwent health examinations in 2003–2004 and 2005–2006 and had no history of HF. Participants were categorized into four groups: proteinuria-free, proteinuria-resolved, proteinuria-developed, and proteinuria-persistent. The outcome of interest was the occurrence of HF. The study included 1,703,651 participants, among whom 17,543 (1.03%) were in the proteinuria-resolved group and 4585 (0.27%) were in the proteinuria-persistent group. After a median follow-up period of 14.04 years (interquartile range 14.19–15.07), HF occurred in 75,064 (4.41%) participants. A multivariable Cox proportional hazards regression analysis indicated that the proteinuria-persistent group had a higher risk of HF compared with the proteinuria-free group (hazard ratio (HR): 2.19, 95% confidence interval (CI): 2.03–2.36, *p* < 0.001). In a further pairwise comparison analysis, participants in the proteinuria-resolved group had a relatively low risk of HF compared with those in the proteinuria-persistent group (HR: 0.64, 95% CI: 0.58–0.70, *p* < 0.001). In conclusion, the risk of HF can change with alterations in the proteinuria status.

## 1. Introduction

Heart failure (HF) is a clinical syndrome characterized by a decline in cardiac contractility, which is often accompanied by impaired ejection of blood from the heart or compromised ventricular filling [1]. This condition is a global health concern, and its prevalence continues to surge worldwide [2]. Despite significant advancements in the development of treatment strategies, morbidity and mortality rates linked to HF remain stubbornly high [2]. Hence, there is a pressing need to comprehensively identify the various risk factors associated with HF. Established risk factors include well-documented contributors such as hypertension, diabetes mellitus, coronary artery occlusive disease, the formation of aortic atheromas, obesity, as well as the consumption of alcohol and tobacco products [3,4,5]. These factors play pivotal roles in the pathogenesis of HF and are crucial targets for intervention. Indeed, by adopting healthier lifestyle habits and diligently managing these cardiovascular risk factors, individuals can significantly reduce their susceptibility to developing HF. However, there remains a critical knowledge gap regarding additional modifiable factors associated with the HF risk.

The acknowledgment of elevated protein levels in the urine, referred to as proteinuria, as a risk factor for cardiovascular diseases and mortality is becoming progressively evident [6,7]. Understanding the mechanisms underlying excessive protein excretion reveals a complex interplay, including heightened glomerular filtration, insufficient tubular absorption or overflow, and augmented secretion. Notably, proteinuria stands out as a significant risk factor for stroke and coronary diseases, independently of other cardiovascular risk factors [8,9]. This multifaceted relationship between proteinuria and adverse cardiovascular outcomes underscores its critical role in the realm of cardiovascular health. Moreover, proteinuria is not limited to its association with immediate cardiovascular risks; it also serves as a harbinger of future disease states, including hypertension, diabetes, and HF [6]. Furthermore, the presence of proteinuria plays a pivotal role in determining the incidence of HF itself [7]. Intriguingly, proteinuria can manifest over time or, conversely, it can be resolved through risk correction or targeted treatment interventions. This dynamic nature of proteinuria suggests that it could indeed be a modifiable factor in the development of heart failure, offering a potential avenue for proactive intervention and prevention. Among the many tests available to measure proteinuria, dipstick testing is commonly used in screening [10]. This test can measure urinary protein exceeding 300 to 500 mg/day. If there are no other factors that can cause false-positive tests (concentrated urine, alkaline urine, hematuria, iodinated contrast agents, exercise, infection, etc.), the dipstick test should be repeated. If the second test result is negative, patients are reassured. However, transient proteinuria is also known to be a risk factor for cardiovascular and cerebrovascular diseases [11,12]. Despite this knowledge, large-scale studies examining changes in the HF risk based on alterations or the persistence of proteinuria remain conspicuously absent.

We hypothesized that the HF risk varies with a change in the proteinuria status. Our aim was to explore the correlation between shifts in the proteinuria status and the risk of heart failure within the context of a comprehensive nationwide, population-based, longitudinal study.

## 2. Materials and Methods

The Korean National Health Insurance System (NHIS) database includes patient-level information about demographics, socioeconomic status, diagnoses, and treatment modalities. Additionally, nationwide health examination data and healthcare institution data are available through the NHIS. NHIS subscribers are recommended to undergo standardized medical health examinations every two years. We included participants from the NHIS–National Health Screening (NHIS-HEALS) cohort. The NHIS-HEALS cohort enrolled participants who underwent medical health screening. We gathered information on their demographics, habits, including the consumption of alcohol, tobacco, regular exercise, income, weight, height, and comorbidities. This study was approved by the Institutional Review Board of the Ewha Womans University Seoul Hospital (approval number: SEUMC 2023-03-017, design of the study: 2 February 2023, IRB approval: 22 March 2023, first draft of the manuscript: 6 May 2023).

**Figure 1:**
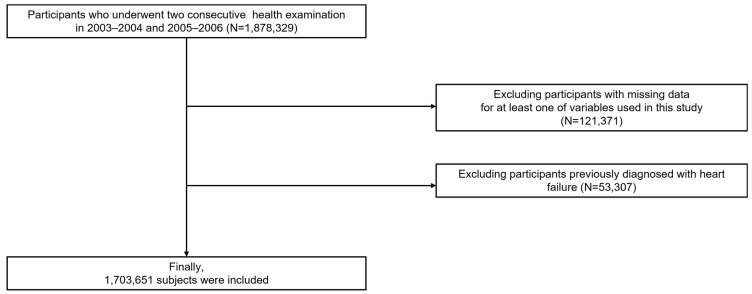
Flow chart of the study participants.

We screened all 1,878,329 individuals who had two repeated health examinations in 2003–2004 and 2005–2006, respectively. Records with absent data for variables of interest (*n* = 121,371) and those with HF (*n* = 53,307) were excluded. A total of 1,703,651 participants were included in the study (Figure 1).

Proteinuria was confirmed by dipstick urinalysis on urine samples collected in the morning after overnight fasting. Dipstick urinalysis measures proteins using Bromphenol blue indicator dye and is most sensitive to albumin. The measurement method is to put midstream urine in a container and wet a test strip. After removing any excess urine from the soaked test strip, the reading is taken approximately one minute later. The value is determined by comparing the strip’s color with a numerical chart, and the results are categorized as follows: negative, 1+ (30 mg/dL), 2+ (100 mg/dL), 3+ (300 mg/dL), and 4+ (>1000 mg/dL). Participants classified the dipstick proteinuria results into two categories: “no proteinuria” and “overt proteinuria (≥ 1+).” Subsequently, study participants were grouped into four categories based on the presence of proteinuria between two consecutive health examinations: (1) “proteinuria-free”, (2) “proteinuria-resolved” (participants with proteinuria at the first screening but not at the second screening), (3) “proteinuria-developed” (participants with developed proteinuria at the second screening), and (4) “proteinuria-persistent”.

The index date corresponded to the health examination date. In cases where individuals underwent multiple examinations between 2005 and 2006, the most recent findings were used for the statistical analysis. The primary outcome of interest was the incidence of heart failure, defined as a participant with a minimum of two claims for HF. It is noteworthy that the diagnostic accuracy of the ICD-10 code (I50) for HF in the NHIS has been rigorously proven and employed in previous research studies [13]. Follow-up was conducted on 31 December 2020 or at the occurrence of HF or death. The study collected various covariates concerning the index date, including age, sex, body mass index (BMI), and family income. Additionally, data on alcohol consumption (frequency per week), tobacco use (none, former, or current), and regular exercise (frequency per week) were gathered through a self-reported questionnaire. Comorbidities were determined using the following criteria. Diabetes mellitus was defined as at least one claim with diagnostic codes (ICD-10 E11–14) with antidiabetic agents or two or more claims with diagnostic codes or a fasting serum glucose level ≥ 7.0 mmol/L or by self-report. Dyslipidemia was defined as at least one claim with diagnostic codes (ICD-10 E78) with related medication or two or more claims with diagnostic codes or a total cholesterol level ≥ 240 mg/dL. Cancer was defined as one or more inpatient claims with diagnostic codes (ICD-10 C00–97) or at least three outpatient claims, along with the specific registration codes ‘V027’ or ‘V193–4’. Renal disease was defined as having two or more claims with diagnostic codes (ICD-10 N17-19, I12-13, E082, E102, E112, E132) or an estimated glomerular filtration rate of less than 60 mL/min/1.73 m^2^. The Charlson Comorbidity Index was calculated as previously described [14,15,16]. For the covariates, findings from the most recent health examinations were applied.

Baseline characteristics were assessed utilizing the Chi-square test for categorical variables and analysis of variance, complemented by the Bonferroni post hoc analysis for continuous variables. To evaluate the association between alterations in the proteinuria status and the incidence of heart failure, Kaplan–Meier survival curves were employed, with statistical significance determined using the log-rank test. Hazard ratios (HRs) were determined using the Cox proportional hazards regression with adjustments for confounding variables. The multivariable Cox regression analysis involved adjusting for the following covariates: model 1, age and sex; model 2, variables in model 1, BMI, income, smoking, alcohol, physical activity, and comorbid diseases (diabetes mellitus, hypertension, dyslipidemia, cancer, and renal disease); model 3, variables in model 2 and the Charlson Comorbidity Index. HRs and 95% confidence intervals (CIs) were used to present the findings of the Cox regression analysis. The assumption of the proportionality of hazards was tested using Schoenfeld residuals, and no violations were detected. A pairwise comparison analysis was used to compare the HF risk among individuals who experienced proteinuria resolution or development. A landmark analysis was performed by excluding participants with HF within one year from the index date. All statistical analyses were conducted using SAS, version 9.2 (SAS Institute, Cary, NC, USA) with the statistical significance indicated by *p*-values < 0.05.

## 3. Results

The study included 1,703,651 participants, among whom 1,661,965 (97.55%) were in the proteinuria-free group, 17,543 (1.03%) were in the proteinuria-resolved group, 19,558 (1.15%) were in the proteinuria-developed group, and 4585 (0.27%) were in the proteinuria-persistent group. The respective second health screenings were performed after a median of 21.5 months (interquartile range, 11.1–25.5 months). The mean age was 43.94 ± 12.05 years, and 69.14% of participants were men. Compared with the proteinuria-free group, the proteinuria-persistent group consisted of older men with higher BMIs and a higher likelihood of comorbidities (Table 1).

The baseline characteristics of the participants according to the heart failure are summarized in Supplementary Table S1. The heart failure group was older, obese, had more frequent consumption of alcohol, and had multiple comorbidities.

**Figure 2:**
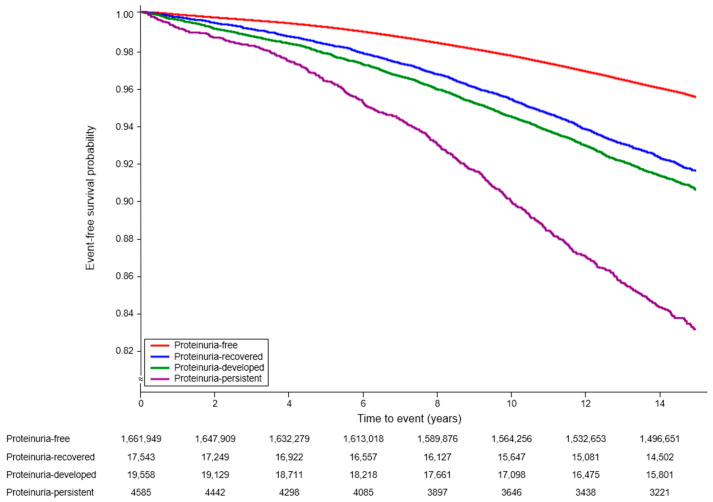
Kaplan–Meier survival curves associated with the proteinuria status and risk of heart failure occurrence.

After a median follow-up period of 14.04 ± 2.36 years, HF occurred in 75,064 (4.41%) participants. Among the proteinuria groups, HF occurred most frequently in the proteinuria-persistent group (Figure 2). The Kaplan–Meier curve showed that there were no significant differences between the proteinuria-resolved group and the proteinuria-developed group. The multivariate analysis indicated that participants in the proteinuria-persistent group had a higher risk of HF than participants in the proteinuria-free group (HR: 2.19, 95% CI: 2.03–2.36, *p* < 0.001, Table 2, model 3) after adjusting for age, sex, and comorbid diseases. Participants in the proteinuria-resolved (HR: 1.31, 95% CI: 1.24–1.38, *p* < 0.001) and proteinuria-developed (HR: 1.52, 95% CI: 1.44–1.59, *p* < 0.001) groups also had a higher risk of HF than those in the proteinuria-free group. In a further pairwise comparison, participants in the proteinuria-resolved group had a lower risk of HF than participants in the proteinuria-persistent group (HR: 0.64, 95% CI: 0.58–0.70, *p* < 0.001). The risk of HF in the proteinuria-developed was higher than that in the proteinuria-free group (HR: 1.52, 95% CI: 1.45–1.60, *p* < 0.001) according to the multivariable analysis (Table 3, model 3). Additionally, when the risk of HF was analyzed according to the degree of proteinuria, the risk of HF was higher in the 4+ proteinuria group than in the proteinuria-negative group (Supplementary Table S2). The risk of HF increased as the degree of proteinuria increased, and this trend was the same in the first (2003–2004) and second (2004–2005) health examinations.

A subgroup analysis performed in regard to the presence of renal disease showed that the risk of HF was higher in the proteinuria-persistent group among participants with and without renal disease (with renal disease, HR: 2.61, 95% CI: 2.22–3.08, *p* < 0.001; without renal disease, HR: 2.05, 95% CI: 1.88–2.23, *p* < 0.001, Supplementary Table S3, Supplementary Figure S1). The proteinuria-resolved (with renal disease, HR: 1.64, 95% CI: 1.39–1.93, *p* < 0.001; without renal disease, HR: 1.27, 95% CI: 1.20–1.35, *p* < 0.001), and proteinuria-developed (with renal disease, HR: 1.47, 95% CI: 1.4–1.55, *p* < 0.001; without renal disease, HR: 2.05, 95% CI: 1.88–2.23, *p* < 0.001) groups also had a higher risk of HF, regardless of the presence of renal disease. The landmark analysis indicated a consistent association between the proteinuria status and the risk of HF (HR: 2.19, 95% CI: 2.03–2.36, *p* < 0.001 in model 3, Supplementary Table S4). The proteinuria-resolved (HR: 1.30, 95% CI: 1.24–1.38, *p* < 0.001 in model 3) and proteinuria-developed groups (HR: 1.51, 95% CI: 1.43–1.58, *p* < 0.001 in model 3) showed an elevated risk of HF compared to the proteinuria-free group.

## 4. Discussion

Our study’s key findings indicate that the HF risk depends on changes in the proteinuria status. The risk of heart failure was notably elevated in cases where proteinuria was newly identified or persistent. Interestingly, we also observed that the risk of heart failure decreased when proteinuria had been resolved.

The presence and severity of proteinuria are strong predictors of the future HF risk, regardless of the estimated glomerular filtration rate or other traditional cardiovascular risk factors [17,18,19]. The Heart Outcomes Prevention Evaluation study was a cohort study with individuals aged 55 or older with cardiovascular disease or its risk factors. After a median 4.5-year follow-up period, microalbuminuria was associated with an increased risk of major cardiovascular events, heart failure, and mortality in patients with and without diabetes mellitus [18]. In a large number (*n* = 10,975) of prospective observational studies of HF-free participants (Atherosclerosis Risk in Communities (ARIC) Study), albuminuria was associated with a future risk of HF [19]. This study categorized the urinary albumin/creatinine ratio as optimal, intermediate normal, high normal, microalbuminuria, and macroalbuminuria. The results showed that the intermediate normal and high normal groups had higher risks of HF compared to the optimal group (adjusted HR, 1.54; 95% CI, 1.12–2.11, adjusted HR, 1.91; 95% CI, 1.38–2.66, respectively). Furthermore, a Japanese atrial fibrillation registry study found proteinuria to be significantly associated with an increased risk of HF among patients with atrial fibrillation [20]. These complementary findings from diverse cohorts emphasize the robustness of the relationship between proteinuria and heart failure, transcending geographical and clinical boundaries. Notably, the presence of proteinuria has consistently emerged as a red flag, signaling an increased susceptibility to various cardiovascular risk factors and the associated mortality risks linked to these factors. Among these risk factors are hypertension, diabetes mellitus, and ischemic heart disease [6]. However, it is imperative to recognize the dynamic nature of the parameters within these cohorts. Our study contributes a fresh perspective to this discourse by revealing a novel finding: the risk of heart failure escalates significantly when proteinuria persists for a duration of at least 2 years. This temporal dimension adds an important layer of insight into the evolving nature of heart failure risk and the potential impact of sustained proteinuria.

In our study, a noteworthy observation emerged: as proteinuria improved, the risk of heart failure (HF) also demonstrated a significant decrease. This finding underscores the potential modifiability of this risk factor, offering a promising avenue for preventive strategies against HF. While there exists a well-established cadre of modifiable risk factors associated with HF, encompassing hypertension, diabetes mellitus, obesity, smoking, and dyslipidemia [21], it is crucial to incorporate proteinuria into the framework of a comprehensive prevention and management strategy for HF. Despite the widely acknowledged significance of proteinuria as a risk factor for HF, relatively few studies have delved into the question of whether the risk of HF diminishes with the improvement of proteinuria. Our research fills this knowledge gap by shedding light on this critical aspect. Intriguingly, our findings revealed that both the group with resolved proteinuria and the group that developed proteinuria had similar risks for HF occurrence. This intriguing revelation underscores the idea that transient proteinuria, which ultimately resolves, may pose a lower risk of HF compared to persistent proteinuria. This shift in risk may, in part, be attributed to the duration of proteinuria (new versus persistent), suggesting that the timeline of proteinuria can influence the risk of HF development. Considering these findings, the potential of correcting proteinuria to reduce the risk of future HF takes on paramount significance.

Although we may not be able to provide an exact mechanism for the association between persistent or improved proteinuria and changes in HF risk, we can propose the following hypotheses. The presence of proteinuria can lead to structural changes in the heart, such as left ventricular hypertrophy [22]. Left ventricular hypertrophy is characterized by the thickening of the walls of the heart’s primary pumping chamber, a response that can occur due to heightened pressure and volume stresses. Left ventricular hypertrophy is associated with an increased risk of HF development. A thickened and stiffened left ventricle may lead to impaired relaxation and filling of the heart, resulting in diastolic dysfunction, which is a common form of HF [23]. Moreover, attenuated proteinuria may reflect improved kidney function and a reduction in the underlying inflammatory processes that contribute to HF development. Additionally, proteinuria-associated conditions, such as hypertension and diabetes, can also increase the risk of HF through various mechanisms, such as the promotion of inflammation, endothelial dysfunction, and oxidative stress [24]. Overall, the association between proteinuria and an increased risk of HF is likely multifactorial, involving both structural changes in the heart and the presence of comorbidities that promote HF development.

Our study had several limitations. Although this was a longitudinal study, it is not possible to confirm a causal relationship with a retrospective cohort study. Since our dataset only included Asian participants, generalization to other ethnicities is difficult. We confirmed proteinuria using a validated dipstick test, but we could not suggest a direct cause of proteinuria. Additionally, the presence of proteinuria was tested by a urine dipstick test instead of through 24 h urine collection. In a former study of the diagnostic accuracy of the urine dipstick test (1+ or higher), the sensitivity was 57.8% and the specificity was 95.4% [25]. A further study using 24 h urine collection is needed to provide stronger evidence of proteinuria improvement and the risk of HF.

## 5. Conclusions

The risk of HF can change with changes in the proteinuria status. Proteinuria can be considered a modifiable risk factor for HF.

## Figures and Tables

**Table 1:** Baseline characteristics of the study population according to the proteinuria status.

Variable	Total	Proteinuria-Free (−/−)	Proteinuria-Resolved (+/−)	Proteinuria-Developed (−/+)	Proteinuria-Persistent (+/+)	*p*-Value
Number of participants (%)	1,703,651	1,661,965 (97.55)	17,543 (1.03)	19,558 (1.15)	4585 (0.27)	
Age, years	43.94 ± 12.05	43.86 ± 12.01	46.74 ± 13.11	46.73 ± 12.99	49.26 ± 12.6	<0.001
Sex						<0.001
Men	1,177,934 (69.14)	1,150,367 (69.22)	11,031 (62.88)	12,928 (66.10)	3608 (78.69)	
Women	525,717 (30.86)	511,598 (30.78)	6512 (37.12)	6630 (33.90)	977 (21.31)	
Body mass index (kg/m^2^)	23.62 ± 3.03	23.61 ± 3.02	24.13 ± 3.32	24.17 ± 3.44	24.94 ± 3.37	<0.001
Household income						<0.001
Q1, lowest	254,366 (14.93)	247,563 (14.90)	3038 (17.32)	3135 (16.03)	630 (13.74)	
Q2	632,196 (37.11)	617,708 (37.17)	6240 (35.57)	6872 (35.14)	1376 (30.01)	
Q3	562,916 (33.04)	549,527 (33.06)	5498 (31.34)	6282 (32.12)	1609 (35.09)	
Q4, highest	254,173 (14.92)	247,167 (14.87)	2767 (15.77)	3269 (16.71)	970 (21.16)	
Smoking						<0.001
Never	980,235 (57.54)	954,998 (57.46)	10,904 (62.16)	11,807 (60.37)	2526 (55.09)	
Former	212,652 (12.48)	207,536 (12.49)	2071 (11.81)	2356 (12.05)	689 (15.03)	
Current	510,764 (29.98)	499,431 (30.05)	4568 (26.04)	5395 (27.58)	1370 (29.88)	
Alcohol consumption (days/week)						<0.001
<3	1,139,835 (66.91)	1,111,388 (66.87)	12,133 (69.16)	13,292 (67.96)	3022 (65.91)	
≥3	563,816 (33.09)	550,577 (33.13)	5410 (30.84)	6266 (32.04)	1563 (34.09)	
Regular exercise (days/week)						<0.001
<3	1,374,142 (80.66)	1341,444 (80.71)	13,597 (77.51)	15,552 (79.52)	3549 (77.40)	
≥3	329,509 (19.34)	320,521 (19.29)	3946 (22.49)	4006 (20.48)	1036 (22.60)	
Comorbidities (%)						
Hypertension	769,339 (45.16)	744,298 (44.78)	10,067 (57.38)	11,373 (58.15)	3601 (78.54)	<0.001
Diabetes mellitus	239,866 (14.08)	228,364 (13.74)	4482 (25.55)	5188 (26.53)	1832 (39.96)	<0.001
Dyslipidemia	421,156 (24.72)	405,688 (24.41)	6250 (35.63)	6871 (35.13)	2347 (51.19)	<0.001
Atrial fibrillation	4448 (0.26)	4234 (0.25)	76 (0.43)	104 (0.53)	34 (0.74)	<0.001
Cancer	31,454 (1.85)	30,290 (1.82)	493 (2.81)	504 (2.58)	167 (3.64)	<0.001
Renal disease	16,806 (0.99)	14,682 (0.88)	785 (4.47)	699 (3.57)	640 (13.96)	<0.001
Charlson Comorbidity Index						<0.001
0	677,492 (39.77)	664,172 (39.96)	5674 (32.34)	6459 (33.02)	1187 (25.89)	
1	691,773 (40.61)	676,615 (40.71)	6467 (36.86)	7314 (37.4)	1377 (30.03)	
≥2	334,386 (19.63)	321,178 (19.33)	5402 (30.79)	5785 (29.58)	2021 (44.08)	
Follow-up duration (years)	14.04 ± 2.36	14.06 ± 2.33	13.56 ± 3.08	13.38 ± 3.33	12.6 ± 3.90	<0.001

Data are presented as the mean ± standard deviation or number (percentage). Q, Quartile.

**Table 2:** Multivariable Cox analysis for the incidence of heart failure according to changes in the proteinuria status.

Proteinuria Status	Total (N)	Heart Failure (N)	IR (per 1000)	HR (95% Confidence Interval)		
				Model 1	Model 2	Model 3
Free	1,661,965	71,276	3.05	1 (ref)	1 (ref)	1 (ref)
Resolved	17,543	1386	5.83	1.93 (1.83, 2.03)	1.32 (1.25, 1.39)	1.31 (1.24, 1.38)
Developed	19,558	1708	6.53	2.16 (2.06, 2.27)	1.53 (1.46, 1.60)	1.52 (1.44, 1.59)
Persistent	4585	694	12.02	4.06 (3.77, 4.38)	2.23 (2.06, 2.40)	2.19 (2.03, 2.36)
*p*-value				<0.001	<0.001	<0.001

Model 1, age and sex. Model 2, variables in model 1, body mass index, income, smoking, alcohol, exercise, history of diabetes mellitus, dyslipidemia, atrial fibrillation, cancer, and renal disease. Model 3, variables in model 2 and the Charlson Comorbidity Index. IR, incidence rate; HR, hazard ratio; CI, confidence interval.

**Table 3:** Pairwise comparison for the incidence of heart failure according to changes in the proteinuria status.

	Model 1			Model 2			Model 3		
	HR	95% CI	*p*-value	HR	95% CI	*p*-value	HR	95% CI	*p*-value
Resolved vs. Free (ref)	1.93	(1.83, 2.03)	<0.001	1.32	(1.26, 1.40)	<0.001	1.31	(1.24, 1.38)	<0.001
Developed vs. Free (ref)	2.17	(2.07, 2.27)	<0.001	1.53	(1.46, 1.60)	<0.001	1.52	(1.45, 1.59)	<0.001
Resolved vs. Persistent (ref)	0.48	(0.44, 0.52)	<0.001	0.64	(0.58, 0.70)	<0.001	0.64	(0.58, 0.70)	<0.001
Developed vs. Persistent (ref)	0.54	(0.49, 0.59)	<0.001	0.73	(0.67, 0.80)	<0.001	0.74	(0.68, 0.81)	<0.001

Model 1, age and sex. Model 2, variables in model 1, body mass index, income, smoking, alcohol, exercise, history of diabetes mellitus, dyslipidemia, atrial fibrillation, cancer, and renal disease. Model 3, variables in model 2 and the Charlson Comorbidity Index; vs., versus; HR, hazard ratio; CI, confidence interval.

## Data Availability

The data that support the findings of this study are available from NHIS-HEALS, but restrictions apply to the availability of these data, which were used under license for the study reported herein and, hence, are not publicly available. Data are, however, available from the authors upon reasonable request and with permission from the National Health Insurance System.

## Supplementary Materials

The following are available online at https://www.mdpi.com/article/10.3390/jpm13121662/s1, Table S1: Baseline characteristics of the study participants according to the heart failure incidence, Table S2: Multivariable Cox analysis for the incidence of heart failure according to the proteinuria significance, Table S3: Multivariable Cox analysis for the incidence of heart failure according to the proteinuria status, Table S4: Multivariable Cox analysis for the incidence of heart failure according to changes in the proteinuria status (landmark analysis), Figure S1: Subgroup analysis for the association between the proteinuria status and heart failure occurrence.
