# Supplementary material for: Resolved Proteinuria May Attenuate the Risk of Heart Failure: A Nationwide Population-Based Cohort Study
Source: J Pers Med. 2023 Nov 28;13(12):1662. doi: 10.3390/jpm13121662 (PMC10744716; doi:10.3390/jpm13121662)

SUPPLEMENTARY MATERIAL

Table S1. Baseline characteristics of the study participants according to heart failure incidence

Variable	Total	Non-heart failure Group	Heart failure Group	p-value
Number of participants (%)	1,703,651	1,628,587 (95.59)	75,064 (4.41)	
Age, years	43.94 ± 12.05	43.36 ± 11.72	56.42 ± 12.21	<0.001
Sex				<0.001
Men	1,177,934 (69.14)	1,131,200 (69.46)	46,734 (62.26)	
Women	525,717 (30.86)	497,387 (30.54)	28,330 (37.74)	
Body mass index (kg/m^2)	23.62 ± 3.03	23.58 ± 3.02	24.54 ± 3.13	<0.001
Household income				<0.001
Q1, lowest	254,366 (14.93)	239,237 (14.69)	15,129 (20.15)	
Q2	632,196 (37.11)	606,831 (37.26)	25,365 (33.79)	
Q3	562,916 (33.04)	540,644 (33.20)	22,272 (29.67)	
Q4, highest	254,173 (14.92)	241,875 (14.85)	12,298 (16.38)	
Smoking				<0.001
Never	980,235 (57.54)	931,602 (57.20)	48,633 (64.79)	
Former	212,652 (12.48)	203,918 (12.52)	8734 (11.64)	
Current	510,764 (29.98)	493,067 (30.28)	17,697 (23.58)	
Alcohol consumption (days/week)				<0.001

<3	1,139,835 (66.91)	1,085 216 (66.64)	54,619 (72.76)	
≥3	563 816 (33.09)	543,371 (33.36)	20,445 (27.24)	
Regular exercise (days/week)				<0.001
<3	1,374,142 (80.66)	1,315,735 (80.79)	58,407 (77.81)	
≥3	329 509 (19.34)	312,852 (19.21)	16,657 (22.19)	
Comorbidities (%)				
Hypertension	769,339 (45.16)	715,526 (43.94)	53,813 (71.69)	<0.001
Diabetes mellitus	239,866 (14.08)	219,385 (13.47)	20,481 (27.28)	<0.001
Dyslipidemia	421,156 (24.72)	390,910 (24.00)	30,246 (40.29)	<0.001
Atrial fibrillation	4448 (0.26)	3035 (0.19)	1413 (1.88)	<0.001
Cancer	31,454 (1.85)	28,618 (1.76)	2836 (3.78)	<0.001
Renal disease	16,806 (0.99)	14,316 (0.88)	2490 (3.32)	<0.001
Charlson Comorbidity Index				<0.001
0	677,492 (39.77)	661,878 (40.64)	15,614 (20.80)	
1	691,773 (40.61)	662,774 (40.70)	28,999 (38.63)	
≥2	334,386 (19.63)	303,935 (18.66)	30,451 (40.57)	
Follow-up duration (years)	14.04 ± 2.36	14.28 ± 1.95	8.87 ± 3.97	<0.001

Q, quartiles.

Table S2. Multivariable Cox analysis for incident heart failure according to proteinuria significance

Proteinuria significance	Total (n)	Heart failure (n)	IR (per 1000)	HR (95% Confidence Interval)		
2005-2006						
				Model 1	Model 2	Model 3
Negative	1679508	72662	3.08	1 (ref)	1 (ref)	1 (ref)
1+	16460	1415	6.4	2.102 (1.994,2.215)	1.478 (1.402,1.558)	1.463 (1.387,1.542)
2+	6175	764	9.58	3.175 (2.957,3.410)	2.000 (1.863,2.149)	1.960 (1.825,2.105)
3+	1261	182	11.59	3.883 (3.358,4.491)	2.576 (2.227,2.979)	2.495 (2.157,2.887)
4+	247	41	13.88	4.673 (3.441,6.347)	3.872 (2.851,5.26)	3.702 (2.725,5.028)
p-value				<.0001	<.0001	<.0001
2003-2004						
Proteinuria significance	Total (n)	Heart failure (n)	IR (per 1000)	HR (95% Confidence Interval)		
				Model 1	Model 2	Model 3
Negative	1681523	72984	3.09	1 (ref)	1 (ref)	1 (ref)
1+	15350	1313	6.33	2.070 (1.960,2.186)	1.416 (1.341,1.496)	1.395 (1.321,1.474)
2+	5519	599	8.24	2.714 (2.505,2.942)	1.715 (1.582,1.859)	1.667 (1.538,1.807)
3+	1115	144	10.42	3.470 (2.947,4.087)	2.116 (1.797,2.492)	2.021 (1.716,2.381)
4+	144	24	13.41	4.509 (3.024,6.724)	3.011 (2.018,4.492)	3.092 (2.072,4.614)
p-value				<.0001	<.0001	<.0001

Model 1 was adjusted for age and sex.

Model 2 was adjusted for age, sex, body mass index, household income, smoking, alcohol consumption, physical activity, history of diabetes mellitus, dyslipidemia, atrial fibrillation, cancer, and renal disease.

Model 3 was adjusted for age, sex, body mass index, household income, smoking, alcohol consumption, physical activity, history of diabetes mellitus, dyslipidemia, atrial fibrillation, cancer, renal disease, and Charlson Comorbidity Index.

IR, incidence rate; HR, hazard ratio; CI, confidence interval;

Table S3. Multivariable Cox analysis for incident heart failure according to proteinuria status

Renal disease					Non-Renal disease				
Proteinuria status	Total (n)	Heart failure (n)	IR (per 1000)	HR (95% CI) Model 3	Proteinuria status	Total (n)	Heart failure (n)	IR (per 1000)	HR (95% CI) Model 3
Free	14682	2008	10.67	1.000 (ref)	Free	1647283	69268	2.99	1.000 (ref)
Resolved	785	154	16.41	1.639 (1.390,1.932)	Resolved	16758	1232	5.39	1.272 (1.202,1.346)
Developed	699	165	21	2.029 (1.729,2.381)	Developed	18859	1543	6.08	1.472 (1.400,1.549)
Persistent	640	163	22.97	2.614 (2.221,3.076)	Persistent	3945	531	10.48	2.045 (1.878,2.228)
p-value				<.0001	p-value				<.0001

Model 1 was adjusted for age and sex.

Model 2 was adjusted for age, sex, body mass index, household income, smoking, alcohol consumption, physical activity, history of diabetes mellitus, dyslipidemia, atrial fibrillation, cancer, and renal disease.

Model 3 was adjusted for age, sex, body mass index, household income, smoking, alcohol consumption, physical activity, history of diabetes mellitus, dyslipidemia, atrial fibrillation, cancer, renal disease, and Charlson Comorbidity Index.

IR, incidence rate; HR, hazard ratio; CI, confidence interval;

Table S4. Multivariable Cox analysis for incident heart failure according to changes in proteinuria status (landmark analysis).

Proteinuria status	Total (n)	Heart failure (n)	IR (per 1000)	HR (95% Confidence Interval)		
				Model 1	Model 2	Model 3
Free	919044	189910	15.55	1 (ref)	1 (ref)	1 (ref)
Resolved	7488	2033	21.39	1.922 (1.822,2.027)	1.320 (1.251,1.393)	1.304 (1.236,1.376)
Developed	8205	2493	24.79	2.148 (2.047,2.255)	1.517 (1.445,1.592)	1.505 (1.434,1.580)
Persistent	986	542	55.22	4.049 (3.756,4.366)	2.226 (2.064,2.401)	2.187 (2.028,2.359)
		p-value		<.0001	<.0001	<.0001

Model 1 was adjusted for age and sex.

Model 2 was adjusted for age, sex, body mass index, household income, smoking, alcohol consumption, physical activity, history of diabetes mellitus, dyslipidemia, atrial fibrillation, cancer, and renal disease.

Model 3 was adjusted for age, sex, body mass index, household income, smoking, alcohol consumption, physical activity, history of diabetes mellitus, dyslipidemia, atrial fibrillation, cancer, renal disease, and Charlson Comorbidity Index.

IR, incidence rate; HR, hazard ratio; CI, confidence interval;

Figure S1. Subgroup analysis for association of proteinuria status and heart failure occurrence

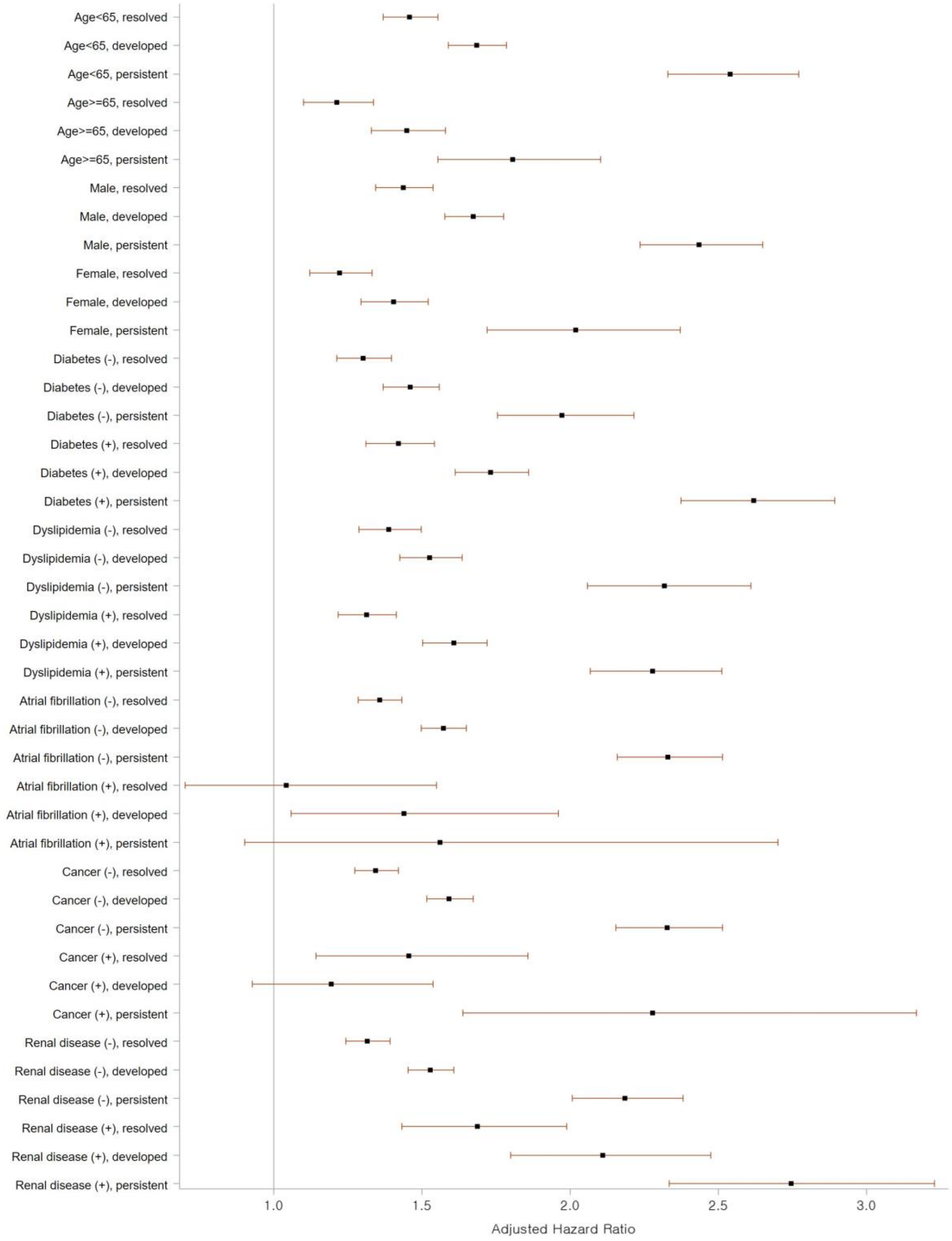

Supplement: Supplementary file 1 [file jpm-13-01662-s001.zip › jpm-2684644-supplementary.pdf]
